# The Impact of a Multidimensional Physical Activity Intervention on Glycemic Control in Type 1 Diabetes: A Preliminary Study

**DOI:** 10.3390/jfmk10020163

**Published:** 2025-05-07

**Authors:** Olga Papale, Emanuel Festino, Francesca Di Rocco, Carl Foster, Iris Prestanti, Sofia Serafini, Pascal Izzicupo, Cristina Cortis, Andrea Fusco

**Affiliations:** 1 Department of Human Sciences, Society and Heath, University of Cassino and Lazio Meridionale, 03042 Cassino, Italy; olga.papale@unicas.it (O.P.); emanuel.festino@unicas.it (E.F.); francesca.dirocco1@unicas.it (F.D.R.); 2 Department of Exercise and Sport Science, University of Wisconsin-La Crosse, La Crosse, WI 54601, USA; cfosteruwl@gmail.com; 3 Department of Medicine and Aging Sciences, University “G. d’Annunzio” of Chieti-Pescara, 66100 Chieti, Italy; iris.prestanti@unich.it (I.P.); sofia.serafini@phd.unich.it (S.S.); pascal.izzicupo@unich.it (P.I.); andrea.fusco@unich.it (A.F.)

**Keywords:** blood glucose, mediterranean diet, sleep quality, chronotype, physical activity

## Abstract

**Objectives**: Type 1 diabetes is characterized by hyperglycemic episodes influenced by diet, sleep quality, chronotype, and physical activity, among others. While aerobic exercise is known to improve glycemic control, its effect on blood glucose regulation remains underexplored. Thus, this case study aimed to evaluate the effects of a prolonged and differentiated indoor and outdoor exercise intervention on glycemic control in an individual with type 1 diabetes. **Methods**: The participant (age: 23 years; weight: 95 kg; height: 1.90 m; Body Mass Index: 26.3 kg/m^2^; waist to hip ratio: 0.98; basal metabolic rate: 2015 kcal; Heart Rate Maximum (HRmax): 197 beats·min^−1^) completed two outdoor (~3800 m) and two indoor sessions with self-selected speed, in the initial 2 min stage, at a 0% grade slope. The grade increased by 2% at each stage during the uphill phase until reaching volitional fatigue, followed by a 2% decrease at each stage during the downhill phase until returning to a 0% grade. Blood pressure was assessed before and after each session. Daily nutrition intake, insulin intake, and blood glucose were continuously monitored. Dietary adherence (PREvención con DIeta MEDiterránea), sleep quality (Pittsburgh Sleep Quality Index), chronotype (Morningness–Eveningness Questionnaire), and physical activity levels (International Physical Activity Questionnaire) were assessed before each session. The Physical Activity Enjoyment Scale was used to measure enjoyment after each session. **Results**: The sessions were completed in ~44 and ~39 min with the participant achieving 84% (outdoor) and 96% (indoor) of their theoretical HRmax. The intervention resulted in glycemic improvements, with time spent in hyperglycemia (>250 mg/dL) decreasing from 56.46% to 0%, while time in the normal range (70–180 mg/dL) increased to 63.96%. A 47% reduction in insulin units showed that insulin sensitivity also improved. **Conclusions**: Despite differences in intensity, indoor and outdoor activities yielded comparable benefits, with outdoor activities being perceived as more enjoyable (outdoor: 28.5 ± 0.7; indoor: 24.0 ± 5.6) and positively impacting glycemic control, thus supporting the need for tailored strategies in diabetes management.

## 1. Introduction

Lifestyle factors, including dietary and physical activity behaviors, are currently targeted to prevent and treat several non-communicable diseases, including diabetes mellitus [1]. Diabetes mellitus is the collective term for heterogeneous metabolic disorders whose primary outcome is chronic hyperglycemia. Hyperglycemia arises from a multifactorial etiology involving genetic, environmental, and behavioral factors and is primarily driven by impaired insulin secretion, impaired insulin action, or a combination of both, with variations across different types of diabetes [2,3]. Diabetes mellitus is the most common metabolic disorder occurring during the lifespan and can be classified into two main types, type 1 and type 2 diabetes. Type 1 diabetes is an autoimmune disorder characterized by the destruction of insulin-producing β cells in the pancreas, leading to absolute insulin deficiency. Common symptoms include polyuria, polydipsia, and weight loss. This metabolic condition disrupts carbohydrate, lipid, and protein metabolism, resulting in acute complications such as diabetic ketoacidosis and chronic complications affecting the cardiovascular, renal, and nervous systems [4,5]. Type 2 diabetes is a metabolic disorder characterized by a state of insulin resistance associated with a progressive loss of β cell function. It is caused by a combination of genetic and biological factors, such as sex and age, and lifestyle factors, including diet, obesity, sedentary behaviors, and consumption of alcohol. This condition is also associated with both acute and chronic complications, including diabetic ketoacidosis, cardiovascular diseases, and diabetic neuropathy [4,5]. Careful control of blood glucose concentration is important for diabetic patients, particularly those with type 1 diabetes mellitus. Regular blood glucose monitoring reduces the risk of hypoglycemia, cardiovascular disease, microvascular and macrovascular complications, and neurological abnormalities. It also helps prevent death, which can result from loss of consciousness and heart failure [6]. Therefore, maintaining blood glucose within safe limits [7] is essential for patients with diabetes.

Blood glucose concentration could be influenced by several factors, such as inadequate duration and poor quality of sleep, both of which increase the risk of impaired glucose regulation and exacerbate diabetes [8]. In fact, sleep duration and quality are important regulators of glucose metabolism. Furthermore, short and long sleep duration, as well as poor sleep quality, have been associated with impaired fasting blood sugar, glucose tolerance, and/or diabetes [8,9]. Sleep restriction activates the sympathetic nervous system, which reduces insulin secretion, promotes insulin resistance, and increases fat accumulation in the liver and muscles. It also alters appetite regulation by lowering leptin levels and raising ghrelin, which increases hunger, especially for carbohydrates. Additionally, reduced physical activity following sleep loss may lead to weight gain [10]. Conversely, excessive sleep may also impair metabolism, influenced by factors such as poor sleep quality, sedentary behavior, and circadian misalignment. It could also reflect comorbidities like depression or hypertension, which are linked to obesity and diabetes, suggesting a potential bidirectional relationship [11].

In addition to sleep, the circadian system could have an influence on glycemic control [8,12,13]. It generates endogenous rhythms of approximately 24 h, the synchronization of which is vital for healthy bodily function. The timing of many physiological processes, including glucose metabolism, is regulated by these rhythms, and disruptions that desynchronize or misalign them can lead to adverse health outcomes. However, the timing of the circadian system varies considerably across individuals, depending on their chronotype [14]. These individual differences, which reflect how subjects assign their sleep and wake times, could influence several activities of daily living [15], including dietary habits and glycemic control. Individuals with a later chronotype tend to exhibit higher glycated hemoglobin (HbA1c) levels and poorer glycemic control, regardless of sleep duration or quality. This association is partially mediated by a greater proportion of daily caloric intake at dinner, suggesting that dietary and circadian mechanisms are involved [16].

Several “healthy” dietary patterns have been associated with improved glucose regulation in diabetes mellitus. Among them, the Mediterranean diet, rich in plant-based foods and low in animal-based, high-fat, and processed foods, has shown significant benefits for preventing and managing diabetes mellitus and reducing the risks of cardiovascular diseases and mortality [1]. Moreover, regular exercise, a key component of the traditional Mediterranean lifestyle, has been shown to positively impact the reduction of the risk of diabetes [17]. The authors of [18] demonstrated that during exercise, whole-body oxygen consumption can increase by as much as 20-fold, with even greater increases occurring in the working muscles. To meet its energy needs under these circumstances, skeletal muscle uses, at a greatly increased rate, its own stores of glycogen and triglycerides, as well as free fatty acids derived from the breakdown of adipose tissue triglycerides and glucose released from the liver [19]. During exercise, hypoglycemia rarely occurs in nondiabetic individuals, as metabolic adjustments, in large part mediated by hormonal mechanisms, help maintain normoglycemia [19]. These hormonal adaptations are essentially lost in insulin-deficient patients with type 1 diabetes. As a consequence, when such individuals have too little levels of insulin in their circulation due to inadequate therapy, an excessive release of counterinsulin hormones during exercise might increase the already high levels of glucose and ketone bodies and could even precipitate diabetic ketoacidosis [19]. Conversely, the presence of high levels of insulin, due to exogenous insulin administration, could attenuate or even prevent the increased mobilization of glucose and other substrates induced by exercise, and hypoglycemia might ensue [18]. In summary, practicing physical activity and exercise can help people with diabetes achieve a variety of goals, including increased cardiorespiratory fitness, increased vigor, improved glycemic control, decreased insulin resistance, improved lipid profile, blood pressure reduction, and maintenance of weight loss. Studies have demonstrated that regular physical activity and/or moderate to high cardiorespiratory fitness are associated with reductions in cardiovascular and overall mortality in people with type 1 diabetes [3,20]. The environmental setting in which physical activity takes place could influence individuals with type 1 diabetes. Outdoor exercise, compared to indoor exercise, has been associated with distinct physiological, psychological, and behavioral responses, including differences in perceived exertion, cardiovascular responses, and enjoyment [21]. These factors could also affect acute glycemic control and long-term adherence to physical activity routines. Therefore, it would be interesting to explore how differences in environmental settings may influence glucose regulation in people with type 1 diabetes.

Given the significant impact of exercise on diabetes management and its long-term effects on glucose metabolism, monitoring physical activity is critical in diabetes care. A multidimensional approach that considers factors beyond glucose levels, such as dietary habits, sleep quality, and chronotype, offers valuable insights for personalized management. The hypothesis tested in this study was that, in individuals with type 1 diabetes, glycemic responses to exercise at varying intensities and settings (indoor vs. outdoor) are influenced not only by the exercise itself but also by lifestyle factors such as sleep quality and chronotype. This case report was designed to provide a detailed, individualized analysis of these interactions in a participant with type 1 diabetes, where individual variability in physiological responses to exercise is particularly relevant. By focusing on a single-case report, we aim to gain insights that can inform future research involving larger cohorts with similar clinical profiles, ultimately contributing to more personalized approaches in disease management. Therefore, this case report aims to evaluate glucose trends in response to different exercise intensities and types, using a multidimensional approach that integrates lifestyle and physiological parameters in a young male participant with type 1 diabetes.

## 2. Materials and Methods

In accordance with the Declaration of Helsinki, the study was approved by the Institutional Review Board of the Department of Human Sciences, Society, and Health of the University of Cassino and Lazio Meridionale (approval number 6663). The study aimed to evaluate the effects of different modalities and exercise intensities on blood glucose regulation in a type 1 diabetes participant. Blood glucose is known to be easily influenced by several internal and external factors, such as insulin and food intake, sedentary lifestyle, or sleep quality. Among these factors, physical activity has been recognized as the most effective. The assessment of the reduction in blood glucose regulation is commonly carried out after generalized strength, resistance, and high-intensity interval training (HIIT) [22,23], aimed only at evaluating the reduction in blood glucose levels. However, to the best of our knowledge, no studies have investigated the effect of a prolonged and differentiated intervention (e.g., incremental walking test and hiking exercise), also focusing on the variations in the self-administered insulin units. Therefore, the present case study proposes a new methodological approach and a new intervention, involving both indoor and outdoor activities, allowing for the evaluation of additional influential variables such as daily nutritional intake, adherence to the Mediterranean diet, sleep patterns, chronotype, and physical activity levels. To establish whether blood glucose regulation could be acutely affected by different exercise modalities, the evaluation of blood glucose concentration was carried out during the entire testing period, from the familiarization session and the enrolment day to the last testing session, during which the external and internal factors were controlled.

### 2.1. Case Description

One 23-year-old male with type 1 diabetes since the age of 14 years took part in the study. The participant was a university student regularly enrolled in a master’s program, with a 10-year history of playing basketball. They stopped practicing regularly due to academic commitments, which led to a more sedentary lifestyle. They were using a multiple daily injection insulin regimen, taking insulin degludec (Tresiba) at bedtime and insulin lispro (Humalog) at mealtimes. The participant had medical clearance to exercise, approved by their medical practitioner, and gave their written consent to participation after receiving both written and oral information regarding the procedures. They were informed that they could withdraw from the study at any time without incurring any adverse consequences.

### 2.2. Procedures

Data collection took place at the Human Performance Lab (HPL) for the indoor sessions and in Gaeta (Lazio, Italy) for the outdoor sessions. The entire testing procedure was conducted in five separate sessions: a familiarization session (enrolment day), two indoor sessions, and two outdoor sessions. Prior to the baseline evaluations, the participant was familiarized with the experimental protocol. To avoid the influence of individual habits on physiological evaluation, the participant was required to refrain from moderate-to-vigorous physical activity and to abstain from alcohol consumption and caffeine intake for at least 24 h before the experimental sessions.

Anthropometric measurements were performed by a certified specialist (i.e., level 2 certification of the International Society for the Advancement of Kinanthropometry). Body weight and height were measured using a scale and stadiometer accurate to 0.1 kg and 0.1 cm, respectively, (Seca, model 709, Vogel & Halke, Hamburg, Germany) and the Body Mass Index (BMI) was calculated. Body circumference measures were made with a tape metric (Holtain Limited, Crymych, UK, 1.5 m Flexible Tape). Waist-to-hip ratio (WHR) was used as a predictor of cardiovascular risk. Waist circumference was measured at the narrowest point in the abdominal region midway between the 10th rib and the crest of the pelvic bone. Hip circumference was measured horizontally at the most prominent points on the posterior, lateral, and anterior sides. The WHR was subsequently computed. To exclude the hypothesis of the presence of cardiovascular diseases, self-monitoring assessment of blood pressure was applied through multiple daily measurements at various time points [24]. The participant was instructed to perform duplicate morning and evening measurements after resting for 5 min and with 1 min between recordings. Two succeeding readings were taken to ensure that blood pressure values did not differ by >5 mmHg from those of a mercury sphygmomanometer (Erkameter 3000; Erka, Bad Tolz, Germany) [24]. Theoretical maximum heart rate (HRmax) was calculated according to the equation [25]:

Theoretical HRmax = 220 − age (years)

Basal metabolic rate was calculated using the Mifflin–St Jeor predictive equation validated for male subjects with diabetes mellitus [26]:

Basal Metabolic Rate = 10 × weight (kg) + 6.25 × height (cm) − 5 × age (years) + 5

In addition, the participant was familiarized with the Borg Category Ratio (CR-10) Rate of Perceived Exertion (RPE) scale, where 0 represented “no exertion at all” and 10 represented “maximal exertion” [27]. During the familiarization session, measurements of glucose history levels, dietary habits, sleep quality, and chronotype (fully described in the following Section 2.2.1 were collected. The same measurement procedure was repeated during all the testing sessions.

After one week, the participant performed two different testing procedures, including two indoor and two outdoor sessions. The indoor sessions consisted of a maximal walking test while the outdoor sessions consisted of a hike. During one indoor and outdoor session, respiratory gas exchanges were continuously monitored using a VO_2_ Master Pro Analyzer (VO_2_ Master Health Sensors Inc., Vernon, BC, Canada). To ensure the reliability and accuracy of the study’s findings, at the beginning of each session temperature and humidity data were recorded during the indoor sessions by maintaining the range of 20–30 °C of temperature and 35–55% humidity, to ensure consistency with the temperature (24.2 ± 1.8 °C) and humidity (42.8 ± 5.5%) of outdoor sessions. Before each exercise session, blood glucose concentrations were also monitored.

The day before and for the entire data collection period (9 days), the participant was asked to take note of their dietary and insulin intake and blood glucose levels.

During the familiarization session and before each session, the participant was asked to complete questionnaires on adherence to the Mediterranean diet, sleep quality, chorotype, and physical activity level. Moreover, blood pressure and recovery index were assessed before each session. The specific parameters evaluated during data collection are described below. After each session, blood pressure, session RPE (sRPE), and enjoyment of the activity were recorded.

During the two indoor testing sessions, the participant underwent a maximal incremental walking test on a treadmill (RunRace HC1200, Technogym, Cesena, Italy), incorporating both uphill and downhill walking. The participant was asked to self-select their speed. The initial speed corresponded to a comfortable walking pace during the first 2 min stage with the slope set at 0%, followed by 2% slope increments every 2 min stage. Once the participant reached the maximum exertion, the uphill phase ended and the downhill phase began, with 2% slope decrements at each stage, until returning to a 0% grade. Heart rate, RPE, and external load (slope %) were monitored throughout the sessions and recorded at the end of each stage. Thirty minutes after each session, sRPE was collected using the CR-10 scale [28].

The two outdoor sessions were carried out in Gaeta (~3800 m in length), Italy. The hiking route was identified based on several parameters such as duration, length, slope, and energy demand according to the Club Alpino Italiano (CAI) Manual. The chosen hike was selected for its similarity to the indoor sessions in terms of duration and mean slope, and it was evaluated according to the walkability principle. The principle considers physical and environmental factors as well as historical and cultural aspects of the route, including the availability of essential services within walking distance, the attractiveness of the route in terms of architecture and social context, and the level of comfort and safety of the route. Thirty minutes after each session, sRPE was asked on a CR-10 scale.

**Figure 1:**
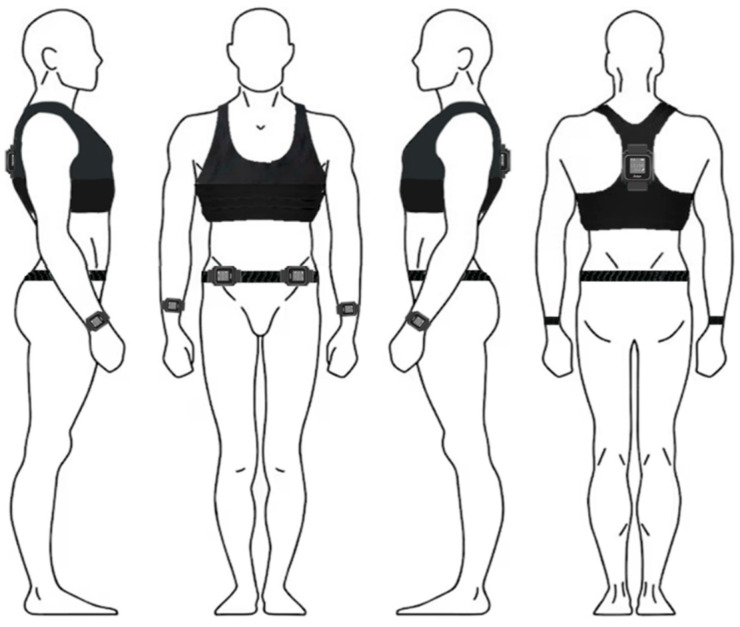
Placement of the Actigraph GT9X accelerometers to evaluate physical activity levels.

To balance methodological rigor with ecological validity, VO_2_ was measured only during two of the four experimental sessions. The remaining sessions were conducted without metabolic equipment to better reflect real-life exercise scenarios. Both indoor and outdoor sessions were performed using the talk test procedure [29], during which the participant was asked to read aloud the standard 40-word paragraph consisting of the Italian version of the Olympic Oath during the last 30 s of each 2 min stage for indoor sessions and every 5 min for outdoor sessions. After reading the paragraph, the participant was asked “Can you speak comfortably?”, with three possible answers: “yes”, which was referred to as a “positive” result; “not sure”, which was referred to as an “equivocal” result; and “no”, which was referred to as a “negative” result. Respiratory gas exchange was assessed using a telemetric open-circuit measurement system (VO_2_ Master Pro Analyzer, VO_2_ Master Health Sensors Inc., Vernon, BC, Canada). The following variables were measured: oxygen uptake (VO_2_), respiratory exchange ratio (RER), and minute ventilation (VE). Additionally, the participant’s physical activity level during the outdoor session was measured under free-living conditions using triaxial accelerometers (GT9X Link; Actigraph, Pensacola, FL, USA) [2,30]. The Actigraph GT9X accelerometers provided information on wear time and daily physical activity (e.g., intensity, number of steps, energy expenditure). According to the literature [31,32], the Actigraph GT9X accelerometers were positioned on both wrists, both ankles, and inside a pouch placed on the posterior torso at the level of the inferior angle of the scapulae (Figure 1). The accelerometers were initialized using the ActiLife6 software (version 6.12.1, ActiGraph, Cary, NC, USA), with a sampling frequency of 30 Hz [33].

#### 2.2.1. Measurements

A multidimensional approach was used to ensure that the four sessions were carried out under consistent conditions and to minimize the influence of external variables on blood glucose levels. During the two indoor and two outdoor sessions, the following parameters were assessed:

Blood Pressure (mmHg): baseline systolic and diastolic blood pressures were measured using a digital sphygmomanometer (Erkameter 3000; Erka, Bad Tolz, Germany). Resting values were obtained after a 15 min period of seated rest, before (PRE) the start of each session. Blood pressure was also measured immediately after the end (POST) and at 15 min (POST-15) and 30 min (POST-30) post-sessions. Post-exercise hypotension was calculated as the difference between PRE and POST-30 values and rate pressure product was calculated by multiplying systolic blood pressure by heart rate [34].

Blood Glucose (mg/dL): blood glucose was collected using a FreeStyle Libre system (Abbott Diabetes Care, Alameda, CA, USA). The device consists of a coin-size sensor and a reader similar to a conventional glucometer. The sensor is applied to the skin with a special applicator and worn for up to 14 days, requiring scans every eight hours to continuously monitor glucose levels. The sensor was connected via Bluetooth to the FreeStyle Libre App, allowing real-time transmission of glucose data. Data were subsequently downloaded using FreeStyle Libre computer software (version 2.12). Blood glucose data were collected alongside blood pressure measurements and throughout the exercise sessions. During indoor sessions, glucose readings were taken at the end of each 2 min stage, while for the outdoor sessions, they were recorded every 5 min.

Recovery Index by Ruffier Test: the standard version of the Ruffier test was administered during the session that included the respiratory gas exchange measurements. According to the literature [21], a modified version of the test was also used, incorporating the talk test procedure. In this variation, the participant was asked to recite the paragraph (fully described in Section 2.2) while performing a 45 s squat exercise. Post-exercise, the participant was asked whether they could speak comfortably, choosing one of three possible responses: “yes” (positive), “not sure” (equivocal), or “no” (negative). Additionally, RPE was recorded. To calculate the Ruffier index, indicative of the individual effort tolerance, recovery capacity, and cardiovascular response [35], the participant’s heart rate was recorded at three given points: at rest (P1), immediately after the 45 s squat exercise (P2) and one minute after the effort (P3). Ruffier index was calculated according to the formula:

Ruffier Index = [(P1 + P2 + P3) − 200]/10

Dietary Measures: to monitor participant eating habits and adherence to the Mediterranean diet, the Italian version of the PREvención con Dieta MEDiterránea (PREDIMED) questionnaire was used [36]. PREDIMED is a 14-item questionnaire in which an adequate consumption of typical traditional Mediterranean foods and low consumption of foods that are not characteristic of the traditional Mediterranean diet adds one point. PREDIMED score was calculated as the sum of all the points attributed to the items. According to the score, adherence was categorized as follows: 0–5, lowest adherence; score 6–9, average adherence; and score ≥ 10, highest adherence. PREDIMED was administered before each session. In addition, daily eating behaviors were monitored the day before and the testing day, using a food diary where the participant recorded the time, type, and quantity of food consumed [37].

Sleep Quality: the Pittsburgh Sleep Quality Index (PSQI) was used to assess the participant’s sleep quality [38]. PSQI is a self-reported questionnaire evaluating subjective sleep quality and disturbances over the previous month. It includes 19 questions grouped into seven components: subjective sleep quality, sleep latency, sleep duration, habitual sleep efficiency, sleep disturbance, use of sleeping medications, and daytime dysfunction. Each component is scored on a scale from 0 to 3, with higher scores indicating greater dysfunction. A total score below 5 is indicative of good sleep quality, while scores of 5 or higher suggest poor sleep quality.

Chronotype: the Morningness–Eveningness Questionnaire (MEQ) is one of the most used in chronobiological and chronopsychological research [39]. It consists of 19 mixed-format items designated to assess an individual’s preferred timing of daily activities, particularly sleep and wake behaviors. The questionnaire includes both Likert-type and time-based questions. Likert-type items offer four options, with lower scores indicating stronger evening preference. Time-based items are scored based on selected time intervals over a 7-h range, with all responses scored from 1 to 5. The total score is the sum of all item scores and it is used to classify chronotype into 5 categories: definitely morning type (70–86), moderately morning type (59–69), neither type (42–58), moderately evening type (31–41), and definitely evening type (16–30).

Physical Activity Level: the Italian short version (7 items) of the International Physical Activity Questionnaire (IPAQ) was administered to evaluate the individual physical activity level [40]. The IPAQ evaluates the frequency, intensity, and duration of physical activity at various levels, namely, low, moderate, and vigorous, along with total physical activity per week. Additionally, it includes an item regarding daily sitting time to estimate sedentary behaviors [41]. The questionnaire includes both categorical and continuous scores. The categorical scores classify participants into three levels, namely, inactive, minimally active, and health-enhancing physical activity, which denotes activity levels exceeding the minimum public health recommendations, which are associated with greater health benefits. The continuous scores are calculated in metabolic equivalent of task (MET) minutes per week.

Enjoyment: After each session, the participant filled in the Physical Activity Enjoyment Scale (PACES) questionnaire to assess satisfaction with the activity [42]. This questionnaire comprises 5 items evaluated on a seven-point Likert scale (AU). For items 1 and 4, the score ranges from 1 (completely agree) to 7 (completely disagree), while for items 2, 3, and 5, the score ranges from 1 (completely disagree) to 7 (completely agree).

Insulin Intake: During the enrolment and on testing days the participant was asked to track the insulin intake using a diary.

**Figure 2:**
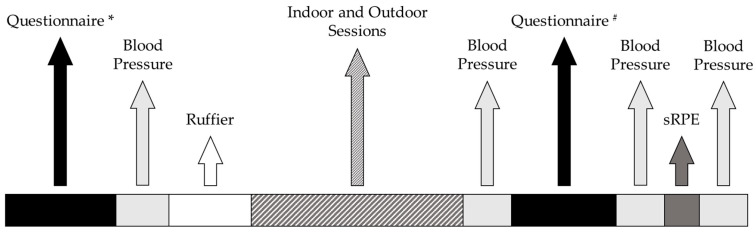
Timeline of the measurements carried out during the experimental sessions. * = PREvención con Dieta MEDiterránea, Pittsburgh Sleep Quality Index, Morningness-Eveningness Questionnaire, International Physical Activity Questionnaire; # = Physical Activity Enjoyment Scale Questionnaire.

Ruffier test, PREDIMED, PSQI, MEQ, and IPAQ were administered before each session. The total duration of each testing session was approximately 2 h. The timeline of the measurements carried out during the experimental sessions is shown in Figure 2.

#### 2.2.2. Data Extraction and Analysis

To quantify the effect of the exercise intervention on blood glucose levels, raw data from the FreeStyle Libre were downloaded using FreeStyle Libre computer software. The analysis included data from the following days: the enrolment day (control session), during which the participant became familiar with the protocol through a verbal explanation; the familiarization session, aimed at accustoming the participant to the procedures; two indoor sessions and two outdoor sessions.

Data were collected between the end of April (indoor sessions) and the beginning of May (outdoor sessions). The period for the outdoor session was chosen according to the mean temperature and humidity collected during the indoor sessions so that they could be replicated during the outdoor sessions. The time interval analyzed was referred to the testing days, from the first measurement of the day (12:04 a.m.) to the last (11:59 p.m.). According to the literature [7,43] ranges of blood glucose levels were identified as follows:

- >250 mg/dL: Very high;
- 181–250 mg/dL: High;
- 180–70 mg/dL: Normal or Target;
- 54–69 mg/dL: Low;
- <54 mg/dL: Very low.

Data were first analyzed as percentage (%) of the total number identified in the different ranges, then converted in time (hours x day). For the Actigraph data, the raw acceleration data on the three axes (x, y, z) were used to obtain the activity counts for each accelerometer, calculated in 60 s epochs using the ActiLife software (version 6.12.1, ActiGraph, Cary, NC, USA), with the low-frequency extension disabled, and vector magnitude units (VMU) activity counts were calculated as:

VMU = √(x^2^ + y^2^ + z^2^)

Energy expenditure and METs were sequentially calculated using algorithms validated by Choi et al. [44] and Freedson et al. [31,45] as follows:

if VMU Counts per Minute were >2453

kcal/min = 0.001064 × VMU + 0.087512 (body mass) − 5.500229

if VMU Counts per Minute were ≤2453

kcal/min = Counts per Minute × 0.0000191 × body mass

Paired *t*-test was used to compare the mean glucose concentration between control and outdoor and indoor sessions. Statistical significance was set at *p* < 0.05.

## 3. Results

The participant weighed 95 kg, had a height of 1.90 m, a BMI of 26.3 kg/m^2^, a WHR of 0.98, a basal metabolic rate of 2015 kcal, an average morning blood pressure of 128–81 mmHg, and an average evening blood pressure of 130–88 mmHg, for systolic—diastolic blood pressure, respectively. Data regarding daily caloric intake, adherence to the Mediterranean diet, sleep quality, chronotype, enjoyment across different sessions, and IPAQ parameters are presented in Table 1.

According to the VO_2_ value achieved during the incremental walking test (45.91 mL/kg/min^−1^), the participant was classified within the “fair” fitness category [46]. To better quantify the overall workload, VO_2_ was also measured during the outdoor session (32.75 mL/kg/min), together with physical activity level data (Table 2).

During the outdoor sessions, the hike lasted ~44 min, with the participant reaching 84% of their theoretical HRmax. In comparison, the indoor sessions lasted ~39 min, during which they reached 96% of their theoretical HRmax (Table 3).

Systolic and diastolic blood pressure, heart rate, post-exercise hypotension, and rate pressure product values across the experimental sessions are shown in Table 4.

**Figure 3:**
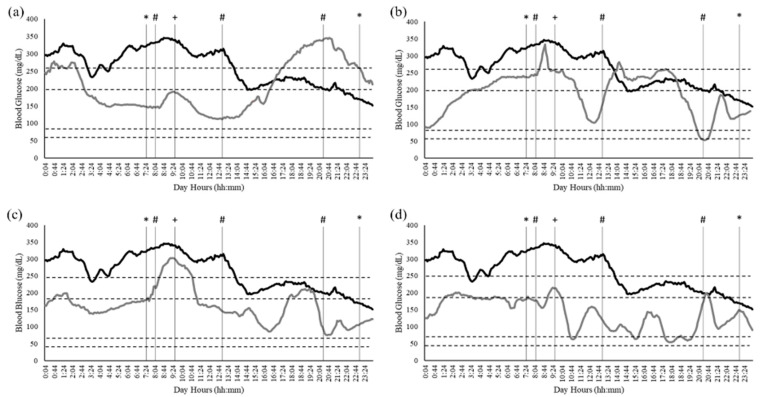
Blood glucose levels in testing days (grey line; (**a**) Indoor Session 1, (**b**) Indoor Session 2, (**c**) Outdoor Session 1, (**d**) Outdoor Session 2) in comparison with the enrolment day (black line). Horizontal dashed lines identify the glucose thresholds and the vertical solid line identifies different time points. * the participant’s wake-up (07:20) and fall asleep (23:10) times; # mealtime and insulin injection; + exercise intervention.

Significant (*p* < 0.0001) differences in blood glucose were found between the enrolment day (261.2 ± 3.2 mg/dL) and both indoor (Indoor 1: 206.2 ± 4.1 mg/dL; Indoor 2: 192.1 ± 3.6 mg/dL) and outdoor (Outdoor 1: 163.1 ± 3.1 mg/dL; Outdoor 2: 137.9 ± 3.6 mg/dL) sessions. The percentage (%) of time spent by the participant within different blood glucose ranges during the analyzed days and the number of units of insulin intake of Humalog (30, 16, 23, 29, 25 units/mL) and Tresiba (34 units/mL) along the data collection are presented Figure 3.

## 4. Discussion

The present study aimed to evaluate the effect of a prolonged exercise intervention on blood glucose regulation in an individual with type 1 diabetes, using a multidimensional approach, in which external variables such as sleep, chronotype, dietary intake, and physical activity levels were monitored. Since type 1 diabetes is a metabolic disease characterized by episodes of hyper- or hypoglycemia which could be influenced by several factors, a multidimensional approach was favored to allow regular control of these parameters, ensuring that the experimental sessions were carried out under consistent conditions. Through this new multidimensional approach, the four sessions were conducted when the participant reported an average/good adherence to the Mediterranean diet [36], a good sleep quality [38], and a chronotype between moderately morning and intermediate [39]. Moreover, in each session, the individual met at least the minimum criteria to be classified as “minimally active” according to the IPAQ [40], while on the enrolment day physical activity levels were lower as confirmed also by high duration in sitting time. The findings of this study demonstrated an improvement in glycemic control in the participant with type 1 diabetes following an intervention that combined aerobic exercise, dietary monitoring, and lifestyle assessment. During the intervention, the participant showed better regulation of blood glucose, with a reduction in % of time spent in hyperglycemic ranges (>250 mg/dL and 181–250 mg/dL) and an increase in the time spent in the target range (70–180 mg/dL). According to the literature [47], reduced time in hyperglycemia may result from enhanced insulin action in muscle and liver, which can be modified by acute and regular bouts of exercise. The literature has shown that aerobic exercise is associated with post-exercise reductions in glycemia in individuals with type 1 diabetes, by improving glucose uptake in skeletal muscle and increasing insulin sensitivity [48]. Riddell and Perkins [49] reported that aerobic exercise, particularly at moderate intensity (30–70% maximum VO_2_), is effective in lowering blood glucose by enhancing glucose utilization. These findings suggest that physical activity, particularly walking both outdoors and indoors, combined with controlled external variables, such as sleep quality, chronotype, and diet, could play a critical role in enhancing glycemic regulation. Walking exercise showed potential benefits for glucose regulation, as reflected by the participant’s self-adjusted insulin doses in response to real-time glucose levels.

The literature reported a fundamental role of diet, sleep, chronotype, and physical activity levels in blood glucose regulation [32,50,51]. Individuals with type 1 diabetes commonly reported poor sleep quality, irregular sleep patterns, and daytime sleepiness, all of which could negatively affect insulin sensitivity and glucose regulation [52]. Similarly, the circadian system plays a key role in glucose metabolism, with variations in chronotype influencing both dietary habits and physical activity levels [16,37,53]. Adherence to the Mediterranean diet, known for its cardiovascular and metabolic benefits, has been associated with improved glycemic control and reductions in several metabolic risk factors, such as body weight, blood pressure, lipid profiles, and HbA1c [50,54]. Additionally, regular exercise, an integral component at the base of the Mediterranean lifestyle pyramid, enhances insulin sensitivity and glucose uptake, contributing to improved glycemic control [19]. Given the combined influence of these variables, adopting a comprehensive and integrated approach is essential for managing diabetes and improving the overall health and well-being of individuals with the condition.

The findings of the present study highlight the acute and chronic effects of aerobic exercise on glycemic control over the four sessions. The participant’s blood glucose levels improved from the enrollment date to the final assessment. The time spent in the blood glucose range >250 mg/dL decreased by 56.46%, while time spent in the range 181–250 mg/dL decreased by 11.67%. These results demonstrate the chronic benefits of aerobic exercise, due to improved insulin sensitivity, and enhanced glucose uptake by skeletal muscles. The improvement over time reflects the potential long-term efficacy of incorporating structured exercise in diabetes management. Moreover, the trend of insulin intake strengthens the benefits of aerobic exercise on both glycemic control and insulin treatment. In particular, a 13-unit decrease in insulin dosage was observed during the first week. This reduction was accompanied by improved glycemic control, with a decrease in time spent in hyperglycemia and an increase in time in the target range (70–180 mg/dL), suggesting acute and chronic improvements in insulin sensitivity due to the increased glucose utilization induced by exercise.

It is interesting to highlight the effect of aerobic exercise performed at different intensities and in different environments on blood glucose control. While the participant achieved on average 84% of their theoretical HRmax during outdoor sessions compared to 96% during indoor sessions, both modalities were effective in reducing time spent in hyperglycemic ranges and increasing time spent in the target range. Despite the lower intensity, outdoor sessions demonstrated comparable benefits in glycemic control and cardiovascular response, as evidenced by similar outcomes in blood glucose, blood pressure, and recovery index. These findings suggest that individuals can achieve effective glycemic improvements from either exercise modality. Those who prefer outdoor activities, such as hiking, can expect similar improvements in insulin action and blood glucose regulation as those who engage in structured indoor exercises. Therefore, in line with our results and the literature [55], which has shown that outdoor exercise is generally perceived as more enjoyable than indoor activities, promoting outdoor exercises may improve adherence to physical activity routines, particularly for diabetic individuals who find indoor exercise less motivating [56]. This could support the long-term maintenance of exercise-related benefits and provide a sustainable strategy to improve blood glucose regulation.

### Limitations and Future Studies

This study presents several limitations that must be acknowledged when interpreting the findings. First, the single-participant case report design limits the generalizability of the results. Although detailed monitoring allowed for an in-depth and individualized analysis, the findings cannot be extrapolated to the broader population of individuals with type 1 diabetes. Moreover, the study did not assess the long-term effects of the intervention. Future studies should investigate whether similar exercise protocols can be sustained over time and contribute to long-term improvements in glycemic control.

In this study, a multidimensional approach was used to evaluate key lifestyle and physiological factors known to influence glycemic control. To this end, several validated instruments were used (e.g., PSQI, PREDIMED, MEQ, IPAQ, dietary intake, and insulin dose management). While these are self-reported measures and may be subject to participant bias, they are widely validated and commonly used in studies involving clinical populations, including individuals with diabetes. Additionally, the participant’s daily caloric intake during the monitored sessions was below the estimated basal metabolic rate. Although this was not an intentional component of the study design, it may have influenced various physiological parameters, including glucose fluctuations, physical performance, and recovery. Future research should consider implementing more rigorous control or monitoring of nutritional intake to minimize potential confounding effects.

Building upon the findings of this single-case study, future investigations should aim to include larger sample sizes to improve external validity. Furthermore, alternative assessment methods should be explored. The digitalization of self-reported measures may enhance data reliability through the use of tools such as actigraphy for sleep, continuous glucose and insulin monitoring systems, and digital food tracking platforms. Finally, future research should aim to confirm the benefits of both indoor and outdoor exercise modalities on glycemic control by including larger and more diverse populations of individuals with type 1 diabetes.

## Figures and Tables

**Table 1:** Summary of daily dietary intake, and lifestyle-related questionnaires (adherence to a Mediterranean diet, sleep quality, chronotype, enjoyment, and IPAQ) results collected across the experimental sessions.

**Daily Dietary Intake**		**Indoor** **Session 1**	**Indoor Session 2**	**Outdoor** **Session 1**	**Outdoor** **Session 2**
CHO (g)		110	196	155	160
PRO (g)		62	72	98	69
FAT (g)		37	24	30	25
Daily Caloric Intake (kcal)		1025	1288	1229	1127
**Questionnaire**	**Enrolment**	**Indoor** **Session 1**	**Indoor** **Session 2**	**Outdoor** **Session 1**	**Outdoor** **Session 2**
Mediterranean Diet (AU)	7	8	7	7	7
Sleep Score (AU)	4	3	2	3	4
Chronotype (AU)	63	62	58	58	57
Enjoyment (AU)	N/A	28	20	28	29
**IPAQ Parameters**					
MET × week (MET)	320	1920	3840	800	1200
Kcal × week (kcal)	1072	4067	8134	2275	2861
Sitting Time (minutes × day)	425	120	150	80	150

CHO: carbohydrates, PRO: proteins; FAT: fats; N/A: not applicable; IPAQ: International Physical Activity Questionnaire; MET: Metabolic Equivalent Task.

**Table 2:** Physical Activity Level data recorded during the outdoor session.

Physical Activity Level	Right Wrist	Left Wrist	Back	Right Waist	Left Waist
METs	6.59	6.57	5.92	3.05	3.32
Energy Expenditure (Kcal)	394	383	375	394	383
Activity counts (counts min^−1^)	3936	3937	3929	4384	3937

METs: Metabolic Equivalent Tasks.

**Table 3:** Psycho-physiological parameters collected during the four experimental sessions.

Psycho-Physiological Parameters	Indoor Session 1	Indoor Session 2	Outdoor Session 1	Outdoor Session 2
Energy Expenditure (kcal)	197	558	553	296
Recovery Index (AU)	1.6	4	3.2	2.8
Mean Heart Rate (beats·min^−1^)	113	126	119	110
Maximum Heart Rate (beats·min^−1^)	196	183	159	170
sRPE (AU)	4	5	4	4

sRPE: session Rating of Perceived Exertion.

**Table 4:** Summary of hemodynamic responses collected across the experimental sessions.

Blood Pressure Measurements		PRE	POST	POST-15	POST-30
Indoor 1	Systolic (mmHg)	131	130	129	130
	Diastolic (mmHg)	88	80	91	86
	Heart Rate (beats·min^−1^)	65	78	75	78
	Post-Exercise Hypotension (mmHg)				1
	Rate pressure product (sBP × HR)				8515
Indoor 2	Systolic (mmHg)	130	129	123	127
	Diastolic (mmHg)	91	92	88	87
	Heart Rate (beats·min^−1^)	65	87	78	82
	Post-Exercise Hypotension (mmHg)				3
	Rate pressure product (sBP × HR)				8547
Outdoor 1	Systolic (mmHg)	131	129	131	128
	Diastolic (mmHg)	79	83	79	77
	Heart Rate (beats·min^−1^)	60	80	68	68
	Post-Exercise Hypotension (mmHg)				3
	Rate pressure product (sBP × HR)				7890
Outdoor 2	Systolic (mmHg)	130	128	121	116
	Diastolic (mmHg)	82	90	84	75
	Heart Rate (beats·min^−1^)	75	91	84	86
	Post-Exercise Hypotension (mmHg)				13
	Rate pressure product (sBP × HR)				9750

sBP: Systolic Blood Pressure, HR: Heart Rate.

## Data Availability

Data available in a publicly accessible repository: The original data presented in the study are openly available at https://github.com/ccortis/Dataset (accessed on 5 May 2025).

## Abbreviations

The following abbreviations are used in this manuscript:

HIIT	High-Intensity Interval Training
WHR	Waist-to-Hip Ratio
RPE	Rate of Perceived Exertion
PREDIMED	PREvención con Dieta MEDiterránea
PSQI	Pittsburgh Sleep Quality Index
IPAQ	International Physical Activity Questionnaire
MeQ	Morningness–Eveningness Questionnaire
sRPE	Session Rate of Perceived Exertion
VO_2_	Oxygen Uptake
CAI	Club Alpino Italiano
RER	Respiratory Exchange Ratio
VE	Minute Ventilation
MET	Metabolic Equivalent Task
PACES	Physical Activity Enjoyment Scale
HbA1c	Glycated hemoglobin
